# Why clinical context and sustainability matters: the role of high-concentration contrast in CEM

**DOI:** 10.1186/s13244-025-02140-0

**Published:** 2025-11-19

**Authors:** Federica Pediconi, Annarita Speranza, Giuliana Moffa, Roberto Maroncelli, Sara Coppola, Francesca Galati, Claudia Bernardi, Giacomo Maccagno, Dominga Pugliese, Carlo Catalano, Andrea Laghi, Veronica Rizzo

**Affiliations:** 1 Department of Radiological, Oncological and Pathological Sciences, Sapienza, University of Rome, Policlinico “Umberto I”, Rome, Italy; 2 Department of Surgical and Medical Sciences and Translational Medicine, Sapienza, University of Rome, Sant’Andrea University Hospital, Rome, Italy

Dear Editor,

We thank Drs. Lobbes and Nelemans for their interest in our article on contrast-enhanced mammography (CEM) using high-concentration iodinated contrast medium (HCCM) [1]. We appreciate their comments, which give us the opportunity to further clarify the scope and interpretation of our work.

First, we would like to emphasize that our study was explicitly designed as a two-center retrospective, exploratory analysis aimed at assessing the diagnostic performance of HCCM in a real-world second-level clinical setting. The intention was not to replace standard contrast protocols or to claim superiority, but rather to demonstrate the feasibility and safety of HCCM as a potentially more sustainable option.

A first criticism of Drs. Lobbes and Nelemans is that our study lacked “a control group with a more standard contrast administration protocol (for example, 1.5 mL/kg with a dose of 300 mg/mL)”. They then cite a publication of Dr. Lobbes himself to support their statement that an injection rate of 1.5 mL/kg with a dose of 300 mg/mL is “standard” [2]. However, it is worth bearing in mind that no dose-finding studies have ever been performed in CEM and that previous studies with low-concentration contrast medium (CM) similarly lacked direct control groups [3]. Current CM injection protocols for CEM are invariably adopted without any comparison of different iodine concentrations, injection rates, or administered dose. We clearly acknowledged in our Discussion that the absence of a direct control group is a limitation of the study, which in part reflects the retrospective nature of the analysis. Nevertheless, we believe our study adds relevant information on the optimization of injection protocols for CEM which currently is very much lacking, as evidenced by Dr. Lobbes’ that “the iodine concentration of the contrast material should vary from 300 mg/mL to 370 mg/mL with a total volume of 1.5 mL/kg of bodyweight (with a maximum of 150 mL) administered at an injection rate of 2–3 mL/s, followed by a saline flush” [2, 4]. Future work could very well focus on comparisons of different iodine concentrations and flow rates, but such studies should have an intra-individual crossover design for clinical relevance, which would raise ethical concerns given the need for two examinations requiring ionizing radiation. In the meantime, comparison of diagnostic performance with literature reports is a widely accepted approach to evaluate clinical utility, and submissions for regulatory approval based on literature evidence are often accepted by authorities for the approval of CM for specific clinical indications (as has been the case for CM approval for CEM applications). Of note is that several recent studies have utilized a 400 mgI/mL iodine concentration (Iomeron-400) at 1 mL/kg bodyweight (i.e., the same low dose protocol utilized in our study), highlighting a growing recognition of the benefits of this protocol (i.e., low iodine dose and high IDR) for CEM [5–7].

A second criticism made by Drs. Lobbes and Nelemans, the study suffered from patient selection and verification bias due in large part to the relatively high prevalence of malignancy in our cohort, which reflected the fact that patients were referred for CEM following initial digital mammography and ultrasound. As clearly stated, the patients included in our study reflect the actual clinical population in which CEM is routinely employed, i.e., patients referred after inconclusive mammography and/or ultrasound, for preoperative staging, or for treatment monitoring. Drs. Lobbes and Nelemans note that “narrowing down a study to a defined subset of patients is necessary to evaluate the diagnostic performance of a test when used for its intended purpose”. In this case, the enrichment of malignant cases is intrinsic to the clinical role of CEM as a problem-solving tool and should not be misinterpreted as selection bias that undermines the validity of our findings. Notably, our diagnostic accuracy estimates are consistent with those of large meta-analyses, further supporting the robustness of our results [8]. As Drs. Lobbes and Nelemans themselves note, “most published studies on diagnostic tests suffer from methodological issues that limit the validity and generalizability of the results”. In our study, histological confirmation or ≥ 12-month follow-up was systematically applied, ensuring appropriate reference standards for diagnostic accuracy assessment. This approach is aligned with accepted methodology in retrospective diagnostic imaging studies.

Finally, we respectfully disagree with the assertion that our conclusions are overstated. Our manuscript cautiously concluded that CEM with HCCM provides “excellent diagnostic performance, while allowing for a reduced iodine dose and increased iodine delivery rate, which may offer a more sustainable alternative without compromising diagnostic accuracy”. This wording was deliberately chosen to reflect both the promise and the limitations of our findings, without generalizing beyond the studied context. Regarding our belief that diagnostic accuracy is not compromised with HCCM despite a reduced iodine dose, it is worth bearing in mind that iodinated CM works by absorbing x-rays and that the contrast effect is directly related to the number of iodine atoms present during image acquisition. In our view, Drs. Lobbes and Nelemans’ assertion that “evidence that the use of the evaluated HCCM-protocol does not compromise diagnostic performance must come from direct comparison with a control group in which a standard-concentration protocol is used” does not take into account basic physics. The aim of our study was precisely to show that a low-dose, high iodine delivery rate protocol using HCCM is at least as effective as the standard injection protocol, thus far promoted [2, 4].

In summary, we believe our study contributes meaningful preliminary evidence that a CM injection protocol utilizing HCCM is feasible and diagnostically robust for routine use in CEM and offers a potentially more sustainable approach to CM use. Our results are intended to stimulate further prospective, controlled trials—an aim that, as highlighted by Drs. Lobbes and Nelemans [2], is shared by the entire breast imaging community.

We appreciate the opportunity to clarify these points and trust that our reply will help readers place our findings in the proper context.

Sincerely, Federica Pediconi (on behalf of all authors)

## References

[1] Pediconi F, Speranza A, Moffa G et al (2025) Contrast-enhanced mammography for breast cancer detection and diagnosis with high concentration iodinated contrast medium. Insights Imaging 16:124. 10.1186/s13244-025-01994-8 40515880 10.1186/s13244-025-01994-8 PMC12167179

[2] Jochelson MS, Lobbes MBI (2021) Contrast-enhanced mammography: state of the art. Radiology 299:36–48. 10.1148/radiol.2021201948 33650905 10.1148/radiol.2021201948 PMC7997616

[3] Pediconi F, Moffa G (2025) Contrast-enhanced mammography: bridging the research gaps and defining the future. Eur J Radiol 192:112351. 10.1016/j.ejrad.2025.112351 40848522 10.1016/j.ejrad.2025.112351

[4] Neeter LMFH, Raat HPJF, Alcantara R et al (2021) Contrast-enhanced mammography: what the radiologist needs to know. BJR Open 3:20210034. 10.1259/bjro.20210034 34877457 10.1259/bjro.20210034 PMC8611680

[5] Bechyna S, Santonocito A, Pötsch N, Clauser P, Helbich TH, Baltzer PAT (2025) Impact of background parenchymal enhancement (BPE) on diagnostic performance of contrast-enhanced mammography (CEM) for breast cancer diagnosis. Eur J Radiol 188:112145. 10.1016/j.ejrad.2025.112145 40318502 10.1016/j.ejrad.2025.112145

[6] Santonocito A, Zarcaro C, Zeitouni L et al (2025) A head-to-head comparison of breast lesion conspicuity at contrast-enhanced mammography and contrast-enhanced MRI. Eur Radiol 35:3070–3079. 10.1007/s00330-024-11195-4 39625504 10.1007/s00330-024-11195-4 PMC12081499

[7] Zarcaro C, Santonocito A, Zeitouni L et al (2025) Inter-reader agreement of the BI-RADS CEM lexicon. Eur Radiol 35:2378–2386. 10.1007/s00330-024-11176-7 39505735 10.1007/s00330-024-11176-7 PMC12021736

[8] Cozzi A, Magni V, Zanardo M, Schiaffino S, Sardanelli F (2022) Contrast-enhanced mammography: a systematic review and meta-analysis of diagnostic performance. Radiology 302:568–581. 10.1148/radiol.211412 34904875 10.1148/radiol.211412

